# Assessment of Volatile Chemical Composition of the Essential Oil of *Jatropha ribifolia* (Pohl) Baill by HS-SPME-GC-MS Using Different Fibers

**DOI:** 10.1155/2013/352606

**Published:** 2013-11-24

**Authors:** Celia Eliane de Lara da Silva, Willian Ferreira da Costa, Sandro Minguzzi, Rogério Cesar de Lara da Silva, Euclésio Simionatto

**Affiliations:** ^1^Centro de Pesquisas e Tecnologia em Recursos Naturais (CPTREN), Pós-Gradução em Recursos Naturais (PGRN), Departamento de Química, Universidade Estadual de Mato Grosso do Sul (UEMS), Rua Emílio Mascolli, 275, 79950-000 Naviraí, MS, Brazil; ^2^Complexos e Centrais de Apoio a Pesquisa (COMCAP), Departamento de Química, Universidade Estadual de Maringá (UEM), Avenida Colombo, 5790, Jd Universitário, 87020-900 Maringá, PR, Brazil

## Abstract

The chemical composition of essential oil and volatile obtained from the roots of *Jatropha ribifolia* (Pohl) Baill was performed in this work. The Clevenger extractor was utilized in hydrodistillation of oil and chemical composition determined by gas chromatography coupled with mass spectrometry detector (GC-MS). The identification of compounds was confirmed by retention index (Kovats index) obtained from a series of straight chain alkanes (C_7_–C_30_) and by comparison with NIST and ADAMS library. A total of 61 compounds were identified in essential oil by GC-MS. The extraction of volatile was performed also by the use of the solid phase microextraction (SPME) with four different fibers. The essential oil extraction was extremely rapid (15 s) to avoid saturation of the fiber and the MS detector. The majority of the composition of essential oil is the terpenes: *β*-pinene (major compound 9.16%), *β*-vatirene (8.34%), *α*-gurjunene (6.98%), *α*-pinene (6.35%), camphene (4.34%), tricyclene (3.79%) and dehydro aromadendrene (3.52%) it and aldehydes and alcohols. Through the SPME it was possible to determine the nine volatile compounds not identified in oil 2,3,4-trimethyl-2-cyclopenten-1-one, *α*-phellandrene, 3-carene, trans-*p*-mentha-2,8-dienol, pinocamphone, D-verbenon, 1,3,3-trimethyl-2-(2-methyl-cyclopropyl)-cyclohexene, 2,4-diisocyanato-1-methylbenzene, and (6-hydroxymethyl-2,3-dimethylehenyl) methanol.

## 1. Introduction

*Jatropha ribifolia* (Pohl) Baill. is a member of *Euphorbiaceae* species found in the semiarid region of northeastern of Brazil [1] and more recently in southeastern [2]. The genus *Jatropha* is constituted of more than 150 species these are the most common in the semiarid region, the *Jatropha mollissima* (Pohl) Baill, *J. mutabilis* (Pohl) Baill and *J. ribifolia* (Pohl) Baill [3]. Other species such as *J. gossypifolia* L. and *J. curcas* L. are found in Brazilian territory and have socioeconomic, medicinal, and ornamental importance [1]. 

The species *J. curcas* can be utilized in biodiesel production to diesel engines [4] and have been targeting very research with catalyst various [5, 6]. The *J. gossypifolia *L. is a plant that can present toxicity but has been used in popular medicine in the treatment of several diseases [7]; however, some compounds may exhibit hepatic toxicity [7, 8]. 

 Many terpene compounds were isolated from the species of *Jatropha* with respect to new chemical structures and medicinal values. These terpenes can exhibit cytotoxic, antitumor, and antimicrobial activities *in vitro*, such as jatrophone, spruceanol, and jatrophatrione view activity against tumor cells [9, 10]. The compounds isolated from are obtained from organic extract such as from bark, stem and root. Some of the terpenes are also isolates the composition of the oil obtained from the fruit. However, the composition of essential oils to volatile terpenes of *Jatropha* species has not been shown.

*J. ribifolia* is aromatic species found in southeastern region also known as “minâncora do campo.” Fernandes et al. [2] newly studying *J. ribifolia* roots, the compounds jatrophone, and cyperenoic acid were isolated from the hexanic extract and characterized by spectroscopic techniques (NMR of ^1^H, ^13^C, and IR). The *in vitro* antiproliferative activity of jatrophone showed selectivity in a concentration-dependent way with Total Inhibition Growth (TGI) of glioma 0.57 *μ*g mL^−1^ (U251), breast cancer 9.2 *μ*g mL^−1^ (MCF-7), adriamycin-resistant ovarian cancer 0.96 *μ*g mL^−1^ (NCI-ADR/RES), kidney 4.2 *μ*g mL^−1^ (786-0), prostate cancer 8.4 *μ*g mL^−1^ (PC-3), colon cancer 16.1 *μ*g mL^−1^ (HT29), and leukemia 0.21 *μ*g mL^−1^ (K-562).

 Extraction of volatile compounds of oil can be performed by purge-trap [11], solid phase extraction (SPE) [12], liquid-liquid extraction (LLE) [13, 14], microwave-assisted hydrodistillation (MAHD) [15, 16], supercritical fluid extraction (SFE) [17], solid phase microextraction (SPME) [18, 19], and by Clevenger extractor [20], among others.

SPME is a technique that has been applied to extraction and concentration of large variety of organic compounds [21, 22] from several types of matrixes such as water [23], air [24], soil [25], volatile components in foods [26, 27], essential oil [18], and biological matrix [28]. Direct determination of analytes through the SPME coupling with other techniques have also been developed [23, 29, 30]. SPME consists of sorbent or adsorbent materials such as polydimethylsiloxane (PDMS), divinylbenzene (DVB), Carbowax (CW), and Carboxen (CAR) dispersed over a silica fiber [22]. In the extraction of volatile compounds it is not necessary to use solvent facilitating the preparation and sample analysis.

This work shows the volatile compounds extraction from essential oil obtained of roots the *J. ribifolia* (Pohl) Baill by using the *headspace* sampling by SPME with different fiber with analyses by mass spectrometry. Chromatography profile obtained by fibers was compared with the chromatography profile of oil direct injection in the same conditions.

## 2. Material and Methods

### 2.1. Plant Material

*Jatropha ribifolia* (Pohl) Bail. roots were collected (3 Kg) in region of grasslands near Navirai-MS city and transported to the organic chemistry laboratory of UEMS-Naviraí. Voucher specimens (JR 0206) have been deposited at the Herbarium of State University of Mato Grosso do Sul (unit Naviraí). The roots were washed in running water to remove earth and dried for 3 hours at room temperature. After mechanical mill was processed and placed in balloon 5 L and essential oil extract with Clevenger extractor apparatus until the exhaustive extraction. The essential oil collected was packaged in glass bottles own, sealed, and stored in the dark for later analysis.

### 2.2. Instrumentation

The chromatographic analyses were carried out using a Thermo-Finigan Gas Chromatograph (Focus DSQ II), equipped with mass spectrometry detector, and a *split/splitless *injector. A fiber holder for manual use was purchased from Supelco (Bellefont). SPME fibers were also from Supelco and coated with four different films: polydimethylsiloxane (PDMS) 100 *μ*m, polyacrylate (PA) 85 *μ*m, polydimethylsiloxane/divinylbenzene (PDMS/DVB) 65 *μ*m and a Carbowax/DVB (CW/DVB) 85 *μ*m. All fibers were conditioned in the hot injector of the gas chromatograph according to instructions provided by the supplier. 

### 2.3. Chromatographic Conditions

Analyses were performed on a gas chromatograph coupled to mass spectrometry (GC-MS Thermo-Finnigan, Focus DSQ II), with a quadrupole mass analyzer, electron impact ionization (70 eV), and autosampler model Triplus. The separation of essential oil and volatile was carried out using a DB-5 capillary column (30 m × 0.25 mm I.D. × 0.25 *μ*m film thickness) with 5% phenyl-methylpolysiloxane. Analytical 5.0 grade helium was used as carrier gas at a flow rate of 1.2 mL min^−1^. The inlet was operated in the *splitless* mode with *splitless* time of 5.0 minute for extraction of volatile by SPME fiber and operated in the *split* mode with injection volume of 2.0 *μ*L the oil diluted in ethyl acetate. The GC temperature program used was 40°C (1 min) and 4°C min^−1^ up to 280°C. The injector, ionization source, and transfer line temperatures were set at 230, 250, and 280°C, respectively. In the TIC mode operation the mass ranged from 50 to 500 amu. Date acquisition was performed by Software Xcalibur 1.4 SR1. Data analysis was performed by NIST MS Search 2.0 library.

### 2.4. HS-SPME Extraction Procedure

The extraction was performed in *headspace* static mode (HS) with all fibers SPME. In addition, to minimize background signals, the fibers are heated in the GC-MS inlet for 5 minutes before each sampling in HS-SPME. Fifty microliters of essential oil were collected and addiction in vials (10 mL) for extraction by SPME enclosed on septum sealed, in laboratory at 25°C. The fibers were exposed by 15 s in *headspace* to volatile extraction to avoid saturation of the fiber coating. The SPME fiber was exposed in the GC-MS injector for 5 min for total desorption of analytes. None carryover was determined to fibers. The dates were collected with Xcalibur software. To analyze the oil pure 50 *μ*L was solubilized in 5 mL of acetyl acetate and injected in to the *split* mode 1/100.

### 2.5. Component Identification

The identification of the volatile components was based on comparison of their mass spectra with those of NIST 2.0 and those described by Adams [31], as well as by comparison of their retention indexes with the literature data [32, 33] and by comparison of their retention times with those of pure authentic samples. 

## 3. Results and Discussion

The roots of *J. ribifolia* were subjected to hydrodistillation for 4 h using a modified Clevenger-type apparatus, with a yield of the 0.09%. The oil showed a green-bluish coloration with a powerful fragrance. To our knowledge, the volatile composition for essential oil of roots has not been shown for *J. ribifolia* in the literature. The complete composition for oil and volatiles was performed through the GC-MS and HS-SPME-GC-MS. The results are viewed in Table 1, where it shows the KI calculated and tabulated to all compounds and its percentage. The main terpenes present in the chemical composition of the root oil of *J. ribifolia *were *β*-pinene (9.16%), *β*-vatirene (8.34%),  *α*-gurjunene (6.98%), *α*-pinene (6.35%), camphene (4.34%), tricyclene (3.79%), and dehydro aromadendrene (3.52%). Alcohols, aldehydes, and ketones represent an important fraction of the chemical composition of the oil, being the compounds *p*-menth-1-en-8-ol (5.24%), 8S-*cis*-5(1H)-azulenone, and 2.4.6.7.8.8a-hexahydro-3.8-dimethyl-4-(1-methylethylidene) (3.33%) the major components with these functional groups.

SPME is a technique of extraction not exhaustive [26], in which an optical fiber coated by sorbent/adsorbent materials is exposed to the *headspace* above the sample or where the fiber is immersed into the aqueous phase. The extraction of analytes can be attributed to their characteristics associated with the fiber coating. Fibers of nonpolar coatings such as PDMS are more suitable for the analysis of nonpolar compounds; PDMS/DVB (bipolar fiber) applied to volatile and nonvolatile low-to-high polarity and polyacrlate (PA) (polar fiber) the volatile compounds extraction of medium to high polarity [22, 34]. For tools fibers the extraction time was short to avoid saturation of the coating of the fiber due to that the oil is highly volatile. Thus there was no saturation of the mass spectrometer detector. A total of sixty-one compounds were identified and compared with the retention KI for oil and volatile composition. The composition of oil evaluated was eighty-seven percent, while for the profile of volatiles it was performed with SPME fiber above ninety-two percent. 

The chromatographic profile obtained from volatile oil with the PDMS and PDMS/DVB fibers were very similar at the beginning of chromatogram where for the tricyclene and *α*-pinene compounds there was no good separation. The CW/DVB and PA generally showed better results in the extraction of low molecular weight compounds as well as for moderately heavy. The compounds of high molecular weight of the oil between 30 and 47 minutes not were extracted by SPME fibers due to the short extraction time or low volatility. 

Through the use of SPME for the volatile extraction it was possible to identify the nine volatile compounds that were not identified by direct injection in GC-MS. These were the (4) 3,3,4-trimethy-2-cyclopentene-1-one, (7) *α*-phellandrene; (11) 3-carene, (13) trans-*p*-mentha-2,8-dienol, (23) pinocamphone, (28) D-verbenon, (29) 1,3,3-trimethyl-2-(2-methyl-cyclopropyl)-cyclohexene, (33) 2,4-diisocyanato-1-methylbenzene, and (39) (6-hydroxymethyl-2,3-dimethylphenyl) methanol. The PA and CW/DVB fibers were selective to extraction of (23) pinocamphone, (33) 2,4-diisocyanato-1-methylbenzene, and (39) (6-hydroxymethyl-2,3-dimethylphenyl) methanol compounds appearing only in these chromatograms. Terpenes are in major concentration to all analyses followed by alcohols/aldehyde and ketones.  Figure 2 shows better this preview.

**Figure 1:**
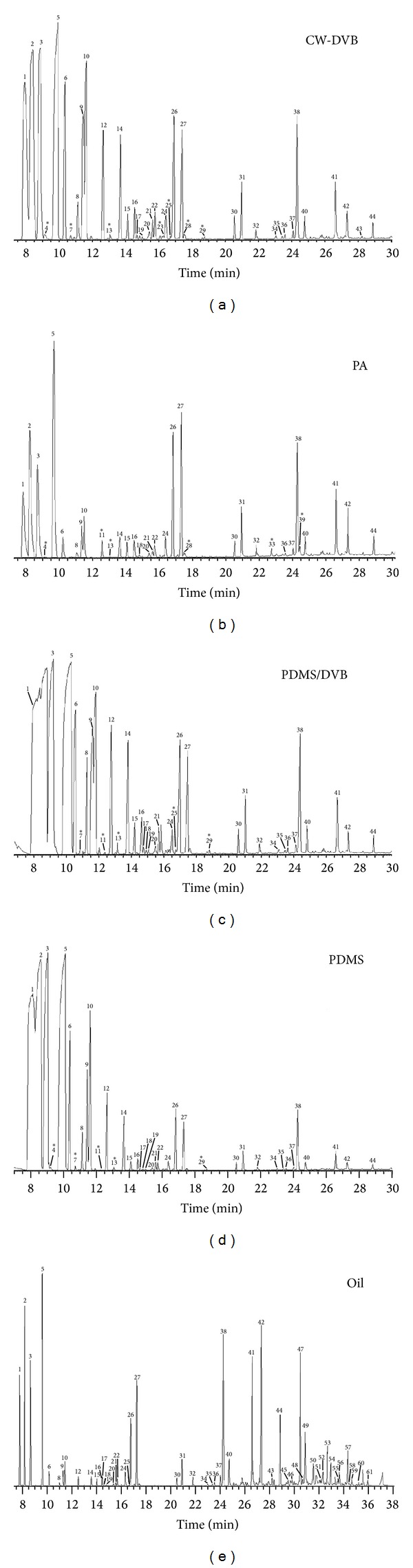
Typical total ion chromatograms (TIC) of *J. ribifolia* oil obtained by GC-MS and volatile compounds determined by HS-SPME-GC-MS **∗** compounds identified only in volatile fraction of oil: (4) 2,3,4-trimethyl-2-cyclopenten-1-one, (7) *α*-phellandrene; (11) 3-carene, (13) trans-*p*-mentha-2,8-dienol, (23) pinocamphone, (28) D-verbenon, (29) 1,3,3-trimethyl-2-(2-methyl-cyclopropyl)-cyclohexene, (33) 2,4-diisocyanato-1-methylbenzene, and (39) (6-hydroxymethyl-2,3-dimethylphenyl) methanol.

**Figure 2:**
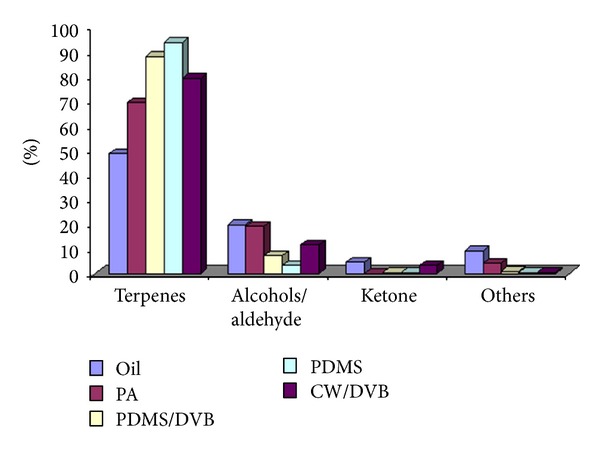
Percentage composition of the major chemical classes detected in the oil and by using different SPME fibers.

**Figure 3:**
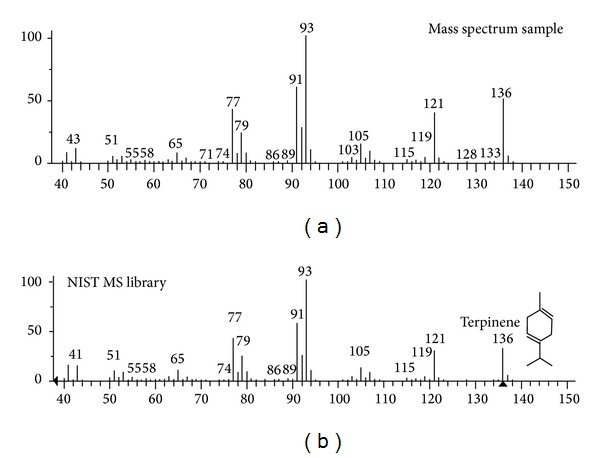
Mass spectrum for *τ*-terpinene obtained from the volatile fraction of the oil by SPME fiber PDMS/DVB and compared to the spectrum of the NIST MS library. Retention time 12.57 min.

Some compounds showed significant differences in the chemical composition of the oil and the chromatographic profile obtained with the fibers. As an example the case of compound  *τ*-terpinene  can be observed. This component is with a low concentration in the oil, while, using the fibers (CW-DVB, PDMS-DVB, and PDMS), this terpene has a higher intensity in the chromatograms (Figure 1). With other monoterpenes such as limonene, *β*-pinene, *α*-pinene, and camphene, among others, this fact also occurs. The compounds were identified by comparing their KI and by mass spectrum in accordance with the example (Figure 3).

## 4. Conclusion

The present study is the first report which describes the volatile chemical composition and essential oil of roots from *J. ribifolia* (Pohl) Baill performed by HS-SPME-GC-MS and GC-MS, respectively. Relative to the plant chemical composition, we conclude that the root's oil is mainly represented by terpenes, alcohols, and aldehyde compounds and that can be used in biological assay. SPME technique showed the profile of chemical composition of volatile to the essential oil. According to the obtained results, it is proposed that in studies involving SPME volatile oils are most effective and safe when fibers are employed with different characteristics, since they can provide different chromatographic profiles of raw oil. Through the analysis procedure performed, it is observed that the fibers were more efficient in extraction of monoterpene compounds meaningful distinction occurring profile obtained for oil and for the fibers obtained, as evidenced in the case of compounds like tricyclene, *α*-pinene, camphene, *β*-pinene, and *β*-myrcene, with a major concentration in the chromatographic profile in SPME fibers. In the case of sesquiterpenes, the raw oil presented a higher concentration of these compounds also showing discrepancy between the profiles of the oil and the fibers used for the analyses. Sesquiterpenes, such as *β*-vatirene, *α*-gurjunene, spathulenol, dehydro-aromadendrene, 6-isopropenyl-4,8a-dimethyl-1,2,3,5,6,7,8,8a-octahydro-naphthalen-2-ol, and 8-oxo-9H-cycloisolongifolene, were significantly more abundant in the chromatographic profile of raw oil. Besides these observations some compounds present the oil in concentrations low (trace levels) which hampered their identification in some SPME fibers. As an example the 6-Isopropenyl-4,8a-dimethyl-1,2,3,5,6,7,8,8a-octahydro-naphthalen-2-ol sesquiterpene with content of 6.64% in oil not was detected by CW/DVB fiber. The same occurs with the phenylpropanoid isoeugenol methylether with a content of 8.54% in oil. 

Due to the possible biological activities for the oil, further studies are in progress in our laboratory to confirm the antiproliferative activity of this plant.

## Figures and Tables

**Table 1:** Chemical composition of oil and volatile the *J. ribifolia* (Pohl) Baill identified by GC/MS and HS-SPME-GC-MS.

		Compounds^a^	KI^b^	KI^c^	Oil	PA	PDMS/DVB	PDMS	CW/DVB
1	7.83	Tricyclene	919	921	3.79	7.52	32.39	21.01	12.26
2	8.24	*α*-Pinene	931	932	6.35	13.19	ND	21.32	15.22
3	8.70	Camphene	945	946	4.34	8.65	11.98	15.15	23.65
4	9.13	2,3,4-Trimethyl-2-cyclopenten-1-one	958	ND	ND	0.01	ND	ND	3.20
5	9.67	*β*-Pinene	974	974	9.16	18.26	19.48	22.3	4.45
6	10.21	*β*-Myrcene	990	988	0.51	1.37	3.71	2.63	0.5
7	10.81	*α*-Phellandrene	994	1002	ND	ND	0.13	0.67	0.01
8	11.06	*α*-Terpinen	1014	1014	0.12	0.15	1.25	0.01	1.01
9	11.35	*p*-Cymene	1022	1022	0.12	0.32	3.06	0.02	0.02
10	11.47	*d*-Limonene	1026	1024	1.55	4.19	6.00	6.33	10.4
11	12.32	3-Carene	1057	1054	ND	ND	0.02	0.01	ND
12	12.57	*t*-Terpinene	1057	1054	0.34	0.87	2.2	1.16	2.4
13	13.05	Trans-*p*-mentha-2,8-dienol	1070	ND	ND	0.10	0.17	0.07	0.17
14	13.63	Terpinolene	1088	1086	0.41	1.23	2.27	1.00	2.56
15	14.12	Linalool	1100	1095	0.33	0.82	0.44	0.17	0.59
16	14.54	*exo*-fenchol	1112	1116	0.42	0.9	0.6	0.25	0.91
17	14.68	Thujone	1116	1114	0.05	ND	0.01	0.01	0.01
18	14.83	cis-*β-*terpineol	1120	1130	0.05	0.18	0.01	0.01	ND
19	14.99	〈*α*-〉campholenal	1125	1126	0.03	ND	0.01	0.02	0.03
20	15.41	*Trans*-pinocarveol	1137	1135	0.03	0.01	0.01	0.02	0.02
21	15.62	(−)-Camphor	1142	1141	0.03	0.01	0.4	0.24	0.01
22	15.76	2,3,3-Timethyl 2-norbornanol	1147	1140	0.05	1.27	ND	0.36	1.2
23	16.20	Pinocamphone	1159	1158	ND	ND	ND	ND	0.02
24	16.36	Borneol	1164	1165	0.76	1.19	0.59	0.024	0.5
25	16.69	3-Pinanone	1173	1172	0.06	ND	0.05	ND	0.77
26	16.80	*p*-Menth-1-en-4-ol, (R)-(−)-	1176	1174	2.91	6.17	2.8	1.14	3.83
27	17.29	*p*-Menth-1-en-8-ol	1185	1179	5.24	7.95	2.39	0.92	3.43
28	17.96	D-verbenone	1209	1204	ND	0.04	ND	ND	0.03
29	18.79	1,3,3-Trimethyl-2-(2-methyl-cyclopropyl)-cyclohexene)	1190	1186	ND	ND	0.09	0.03	0.02
30	20.55	2,6-Octadienoic acid, 3,7-dimethyl-, ethyl ester	1195	1194	0.36	0.61	0.32	0.12	0.01
31	20.94	Cyclopropane, Trimethy(2-methyl-1-propenylidene)-	1285	ND	1.23	2.06	0.82	0.3	1.05
32	21.83	Methyl geranate	1296	1322	0.46	0.60	0.18	0.07	0.22
33	22.75	2,4-Diisocyanato-1-methylbenzene	1301	ND	ND	0.38	ND	ND	ND
34	23.04	Isolongifolene, 9,10-dehydro-	1361	ND	0.12	ND	0.07	0.03	0.05
35	23.43	*β*-Vatirene	1312	ND	8.34	ND	0.13	0.02	0.02
36	23.59	*β*-Patchoulene	1324	1322	0.26	0.27	0.02	0.01	0.15
37	24.05	*β*-Elemene-(−)	1361	1391	0.01	0.13	0.05	0.02	0.05
38	24.30	Cyperene	1373	1398	0.01	6.45	2.72	1.19	3.35
39	24.46	(6-Hydroxymethyl-2,3-dimethylphenyl) methanol	1378	1379	ND	0.01	ND	ND	ND
40	24.77	Isoledene	1393	1389	1.35	0.89	0.36	0.14	0.44
41	26.6	*α*-Gurjunene	1400	1409	6.98	3.05	0.93	0.34	1.18
42	27.36	Isoeugenol methylether	1494	ND	8.54	2.41	0.4	0.22	ND
43	28.23	Spathulenol	1514	1490	0.74	ND	ND	ND	0.09
44	28.87	Aromadendrene, dehydro-	1519	ND	3.52	0.93	0.22	0.11	0.32
45	28.38	Corimbolone	1533	ND	0.01	ND	ND	ND	ND
46	29.72	isolongifolene-5-ol	1534	ND	0.65	ND	ND	ND	ND
47	30.52	4,4,11,11-Tetramethyl-7-tetracyclo [6.2.1.0(3.8)0(3.9)]undecanol	1608	ND	6.64	0.39	0.15	0.14	ND
48	30.83	Cedrol	1619	ND	0.01	ND	ND	ND	ND
49	30.91	8S-*cis*-5(1H)-Azulenone, 2,4,6,7,8,8a-hexahydro-3,8-dimethyl-4-(1-methylethylidene)	1622	ND	3.33	ND	ND	ND	ND
50	31.55	Tujopsanone 〈3-〉	1645	1650	1.25	ND	ND	ND	ND
51	32.0	4a,8,8-Trimethyloctahydro cyclopropa(d)naphthalen-2(3H)-one	1661	ND	0.01	ND	ND	ND	ND
**52**	32.34	(−)-Spathulenol	1673	ND	1.25	ND	ND	ND	ND
**53**	32.69	6-Isopropenyl-4,8a-dimethyl-1,2,3,5,6,7,8,8a-octahydro-naphthalen-2-ol	1696	ND	1.15	ND	ND	ND	ND
**54**	32.90	*β*-Guaiene	1697	ND	0.02	ND	ND	ND	ND
**55**	33.41	2,2,7,7-Tetramethyltricyclo [6.2.1.0(1,6)]undec-4-en-3-one	1713	ND	0.46	ND	ND	ND	ND
**56**	33,92	2(1H)naphthalenone, 3,5,6,7,8,8a-hexahydro-4,8a-dimethyl-6-(1-methylethenyl)	1732	ND	1.15	ND	ND	ND	ND
**57**	34.12	Acetic acid, 7-isoprophenyl-1,4a-dimethyl-3-oxo-2,3,4,4a,5,6,7,8-octahydronaphthalen-2-il esther	1740	ND	0.09	ND	ND	ND	ND
58	34.16	Oxide-[2] aromadendrene	1762	ND	0.01	ND	ND	ND	ND
59	34.78	8-Oxo-9H-cycloisolongipholene	1765	ND	2.09	ND	ND	ND	ND
60	35.08	Valeral	1776	ND	0.41	ND	ND	ND	ND
61	35.94	Methyl hinokiate	1827	ND	1.20	ND	ND	ND	ND

Total (%)					87.06	92.58	96.43	97.58	94.15
Terpenes					48.51	69.53	87.9	93.8	79.11
Alcohols/aldehyde					19.66	18.99	7.22	3.124	11.54
Ketone					4.66	0.06	0.41	0.25	3.27
Others					14.23	4.00	0.90	0.41	0.23

RT: retention time; ND: not determined; ^a^compounds listed in order of elution from a DB-5 column; ^b^KI calculated; ^c^KI tabulated. PA: polyacrylate fiber; PDMS/DVB: polydimethylsiloxane/divinylbenzene fiber; PDMS: polydimethylsiloxane fiber; CW/DVB: Carbowax/divinylbenzene fiber.
